# Multidimensional Inflammatory Biomarker Profiling in COPD: A Latent Inflammatory Construct Associated with Disease Severity and Exacerbation Burden

**DOI:** 10.3390/diagnostics16193161

**Published:** 2026-09-29

**Authors:** Oana Maria Catana, Ramona Cioboata, Denisa Maria Mitroi, Ovidiu Mircea Zlatian, Mircea Popescu Driga, Costin Teodor Streba

**Affiliations:** 1 Doctoral School, University of Medicine and Pharmacy, 200349 Craiova, Romania; oana_cattana@yahoo.com (O.M.C.); denisa_maria2@yahoo.com (D.M.M.); 2 Pneumology Department, University of Medicine and Pharmacy, 200349 Craiova, Romania; costin.streba@umfcv.ro; 3 Microbiology Department, University of Medicine and Pharmacy, 200349 Craiova, Romania; ovidiu.zlatian@umfcv.ro; 4 Immunology Department, University of Medicine and Pharmacy, 200349 Craiova, Romania; mirceapopescu100@gmail.com; 5 Centre for Research in Gastroenterology and Hepatology (CCGH), 200349 Craiova, Romania

**Keywords:** chronic obstructive pulmonary disease, systemic inflammation, exacerbation burden, disease severity, interleukin-8, tumour necrosis factor-α

## Abstract

**Background/Objectives**: Systemic inflammation is a widely recognised feature of chronic obstructive pulmonary disease (COPD). The combined contribution of acute-phase reactants and pro-inflammatory cytokines to disease severity and exacerbation risk remains incompletely understood. This study evaluated the associations of TNF-α, IL-8, C-reactive protein (CRP), erythrocyte sedimentation rate (ESR), and fibrinogen with clinical outcomes in COPD and investigated whether a latent systemic inflammation construct provides additional information on exacerbation burden beyond individual biomarkers. **Methods**: We conducted a cross-sectional observational study in Romania, including 96 patients with clinically stable COPD. TNF-α and IL-8 were measured by ELISA, whereas CRP, ESR, and fibrinogen were determined using standard laboratory methods. Spirometry and patient-reported outcomes were assessed, and exacerbation history over the preceding 12 months was recorded retrospectively via patient interviews and verified against medical records when available. Negative binomial regression, Cox proportional hazards modelling, and generalised structural equation modelling (GSEM) were used to examine associations between inflammatory biomarkers and clinical outcomes. **Results**: All five inflammatory biomarkers increased across GOLD grades and correlated inversely with spirometric indices, with ESR showing the strongest correlations. Higher biomarker concentrations were associated with worse CAT, mMRC, and SGRQ-C scores (all *p* < 0.001). Individual biomarkers were associated with exacerbation frequency in unadjusted analyses but lost significance after adjustment for disease severity and comorbidities. In contrast, the latent systemic inflammation construct remained independently associated with annual exacerbation rate (IRR = 1.407, 95% CI: 1.145–1.668, *p* < 0.001). Neither the individual biomarkers nor the eosinophilic phenotype showed a clinically meaningful association with time to first exacerbation. **Conclusions**: A latent systemic inflammation construct integrating multiple biomarkers remained independently associated with exacerbation burden after additional adjustment for GOLD spirometric grade, although the association was attenuated (IRR = 1.186 vs. 1.407 in the primary model) and should be interpreted as exploratory given the differing covariate adjustment sets. These findings support further investigation of integrated inflammatory profiling in COPD, particularly in prospective multicentre studies.

## 1. Introduction

Over the past two decades, chronic obstructive pulmonary disease (COPD) has evolved from a condition characterised primarily by irreversible airflow obstruction into a complex multisystem disease with substantial extrapulmonary involvement [1]. In spite of advances in prevention and treatment, COPD continues to be a leading cause of morbidity and mortality internationally [2], imposing a substantial clinical, social, and economic burden [3].

Persistent exposure to cigarette smoke and other inhaled irritants initiates an inflammatory response involving both innate and adaptive immune cells, including airway epithelial cells, alveolar macrophages, neutrophils, and lymphocytes, along with the release of numerous cytokines and chemokines [4]. In many patients, this response is accompanied by systemic inflammation, which has been linked to disease progression and the increasing burden of COPD-related comorbidities [5]. However, systemic inflammation is not uniform across patients, and its variability has been proposed as a mechanism underlying the clinical heterogeneity of COPD, including differences in disease severity, recurrent exacerbations, and prognosis [6].

The exacerbation burden is one of the clearest clinical expressions of this heterogeneity. Acute exacerbations are a defining feature of COPD and a major determinant of disease progression, with frequent exacerbators showing a steeper decline in FEV_1_, lower health-related quality of life, higher hospitalisation rates, and increased mortality compared with those with infrequent events [7]. Frequent exacerbators also tend to show higher levels of systemic inflammatory activity, pointing to a possible biological substrate for this more aggressive clinical course [8].

Although exacerbation history and spirometric assessment remain central to risk stratification, they provide limited insight into the biological mechanisms underlying clinical diversity [9]. Among the numerous inflammatory mediators implicated in COPD pathogenesis, tumour necrosis factor-α (TNF-α) and interleukin-8 (IL-8) have emerged as key cytokines because of their central roles in amplifying systemic inflammation and sustaining neutrophil-driven immune responses [10,11]. Their activity is accompanied by changes in established inflammatory markers, such as C-reactive protein (CRP), erythrocyte sedimentation rate (ESR), and fibrinogen, which together provide complementary information on the systemic inflammatory profile in COPD.

CRP, ESR, and fibrinogen, although less specific than cytokine-level markers, remain attractive candidates because of their low cost and widespread clinical availability. CRP is the most extensively studied acute-phase reactant in COPD, with elevated concentrations correlating with reduced lung function and increased exacerbation risk [12]. Fibrinogen has been independently associated with reduced pulmonary function and increased COPD risk [13], and, together with CRP and leukocyte count, contributes to prognostic algorithms for exacerbation risk [12]. ESR, a nonspecific but widely available marker, correlates less consistently with COPD severity than CRP when examined individually, although combined elevation of ESR and CRP has been linked to more pronounced systemic derangement [14].

Tumour necrosis factor-α (TNF-α) is a pro-inflammatory cytokine produced predominantly by activated macrophages, monocytes, and T lymphocytes [10] and is considered one of the principal mediators of the immune response in COPD [15]. TNF-α amplifies downstream inflammatory pathways, promotes oxidative stress, and contributes to tissue remodelling [10], while its activation of NF-κB also drives skeletal muscle catabolism, linking pulmonary inflammation to systemic manifestations of the disease [16]. Elevated circulating TNF-α concentrations have been associated with reduced lung function, muscle wasting, metabolic dysfunction, and an increased exacerbation risk, although the strength of these associations has varied across clinical studies [15]. The limited clinical efficacy of anti-TNF therapies [17] indicates that TNF-α functions as a single component within a multifaceted inflammatory network, rather than serving as the sole driver of COPD pathogenesis [16].

Interleukin-8 (IL-8), also referred to as CXCL8, is a chemokine that plays an essential role in the granulocyte-driven inflammatory response characteristics of COPD. It is released primarily by airway epithelial cells, alveolar macrophages, and activated immune cells in response to inflammatory stimuli, promoting neutrophil recruitment and activation within the airways [11]. Persistent IL-8 expression sustains this process, alongside protease release, oxidative stress, and progressive tissue destruction, thereby contributing to airflow limitation and structural lung damage typical of COPD [11]. Increased IL-8 concentrations have been reported in both the airways and the systemic circulation of patients with COPD [18], particularly during acute exacerbations, and several studies have linked higher circulating levels to increased exacerbation frequency and more advanced disease [19]. These observations indicate that IL-8 may provide clinically relevant information beyond conventional markers, although its utility as a biomarker of COPD progression remains to be fully established [20]. Upstream cytokines such as IL-17 have also been increasingly implicated in neutrophilic airway inflammation and exacerbation susceptibility, highlighting the complexity of the inflammatory pathways underlying COPD [21,22]. Although IL-17 was not measured in the present study, its role provides additional biological context for the selection of IL-8 as a marker of neutrophilic inflammation.

TNF-α and IL-8 were selected as primary markers of interest because they capture distinct aspects of the inflammatory response in COPD: TNF-α drives systemic inflammation and its metabolic consequences [10,15], while IL-8 sustains neutrophil recruitment and the persistence of airway inflammation [11]. Most biomarker studies have evaluated inflammatory markers individually. Composite approaches have been explored to a limited extent: simultaneous elevation of CRP, fibrinogen, and leukocyte count was associated with increased exacerbation risk in a large population-based study [23], consistent with earlier findings from the Bergen COPD Cohort Study, in which elevated CRP was associated with exacerbation frequency [24]. Structural equation modelling has also been applied to characterise systemic manifestations of COPD in smokers, supporting the broader premise that latent-variable approaches can capture shared inflammatory or systemic signal beyond individual markers [25,26].

To our knowledge, no published study has specifically applied latent-variable modelling to the combined assessment of acute-phase reactants (CRP, ESR, fibrinogen) and these pro-inflammatory cytokines (TNF-α and IL-8) to determine whether their shared variance provides information beyond that captured by individual biomarkers. The present study therefore aimed to characterise the systemic inflammatory profile of patients with stable COPD, to examine the individual and joint associations of TNF-α, IL-8, CRP, ESR, and fibrinogen with disease severity and exacerbation burden, and to determine whether their integrated inflammatory profile is independently associated with exacerbation frequency.

## 2. Materials and Methods

### 2.1. Study Design and Population

This cross-sectional observational study investigated the relationships between individual inflammatory biomarkers and an integrated systemic inflammatory profile with disease severity and exacerbation burden in patients with stable COPD. Circulating TNF-α, IL-8, CRP, ESR, and fibrinogen were evaluated individually and in combination to characterise their contribution to clinical out-comes.

The study included 96 patients with a confirmed COPD diagnosis admitted to the Department of Pneumology at “Victor Babes” Clinical Hospital of Infectious Diseases and Pneumophthisiology in Craiova, Romania, between January and December 2025. Only clinically stable patients were enrolled, with no acute exacerbation during the four weeks before enrolment. Participants attended the department for scheduled clinical evaluation, pulmonary function testing, and optimisation of maintenance therapy.

The study was conducted in accordance with the principles of the Declaration of Helsinki and was approved by the Ethics Committees of the University of Medicine and Pharmacy of Craiova (Approval No. 407/20.11.2024) and the “Victor Babes” Clinical Hospital of Infectious Diseases and Pneumophthisiology, Craiova (Approval No. 15524/30.10.2024). Written informed consent was obtained from all participants before enrolment.

### 2.2. Inclusion and Exclusion Criteria

Eligible participants were 40–80 years of age, had a confirmed diagnosis of COPD based on a post-bronchodilator FEV_1_/FVC ratio < 0.70 according to the 2025 GOLD criteria [2], and provided written informed consent. Both ever-smokers and never-smokers were eligible, given the recognised contribution of non-tobacco exposures to COPD in this region [27]. Patients with common cardiovascular and metabolic comorbidities, including arterial hypertension, type 2 diabetes mellitus, heart failure, obstructive sleep apnoea syndrome, obesity, and atrial fibrillation, were eligible for inclusion because they represent common COPD-related comorbidities encountered in routine clinical practice [28,29]. A previous history of successfully treated pulmonary tuberculosis was not considered an exclusion criterion given its relatively high regional prevalence [30].

Patients were excluded if they were younger than 40 years or older than 80 years, had chronic respiratory diseases other than COPD, an active malignancy, or severe hepatic or renal disease, had received immunomodulatory therapy or systemic corticosteroids for more than four weeks during the six months pre-ceding enrolment, had experienced an acute COPD exacerbation within four weeks before enrolment, or declined to provide written informed consent. Active malignancy was exclusionary given the independent pro-inflammatory effects of malignant disease on circulating cytokine concentrations [31]. Severe hepatic or renal disease was similarly excluded owing to its known influence on the hepatic synthesis and clearance of acute-phase reactants, including CRP and fibrinogen [32]. Long-term systemic corticosteroid use was excluded be-cause of the well-established suppressive effects of glucocorticoids on circulating inflammatory markers, including TNF-α and IL-8 [33]. Only participants with complete clinical, spirometric, and laboratory data were included in the final analysis.

### 2.3. Demographic, Anthropometric, and Clinical Assessment

Baseline demographic and clinical data were obtained from patient interviews and verified using hospital medical records. Recorded variables were age, sex, place of residence (rural or urban), smoking status (current or former smoker), age at smoking initiation and smoking duration (years). Body weight and height were measured at enrolment, and body mass index (BMI) was calculated and expressed as kg/m^2^.

Clinical assessment included time since COPD diagnosis, the number of acute exacerbations during the preceding 12 months (requiring outpatient treatment or hospitalisation) and the presence of chronic respiratory symptoms, including cough and dyspnoea. Dyspnoea severity was assessed using the modified Medical Research Council (mMRC) dyspnoea scale [34]. Disease-specific health status was assessed with the COPD Assessment Test (CAT) [35] and the COPD-specific St George’s Respiratory Questionnaire (SGRQ-C) [36].

Maintenance COPD therapy was recorded, including LABA monotherapy, LABA/LAMA, ICS/LABA, triple inhaled therapy (LABA/LAMA/ICS) and long-term oxygen therapy (LTOT) when applicable. Relevant comorbidities, such as arterial hypertension, type 2 diabetes mellitus, heart failure, atrial fibrillation, obesity, obstructive sleep apnoea syndrome, and a history of treated pulmonary tuberculosis, were also documented.

### 2.4. Pulmonary Function Assessment

Pulmonary function was assessed by post-bronchodilator spirometry during the baseline evaluation to confirm airflow obstruction and assess disease severity. All measurements were obtained using calibrated equipment (Spirolab IV spirometer—Medical International Research, Rome, Italy) and performed by trained respiratory technicians, following ATS/ERS standardisation guidelines [37].

Recorded spirometric parameters included forced expiratory volume in one second (FEV_1_), forced vital capacity (FVC), the FEV_1_/FVC ratio (Tiffeneau index), and forced expiratory flow between 25% and 75% of the forced vital capacity (FEF_25–75_%), expressed as absolute values and percentages of the predicted values [38].

A post-bronchodilator FEV_1_/FVC ratio below 0.70 was used to confirm the presence of persistent airflow obstruction, as recommended by the 2025 GOLD guidelines [2]. Patients were subsequently classified into GOLD spirometric grades based on FEV_1_ (%predicted).

### 2.5. Assessment of Exacerbation Burden

Because all patients were clinically stable at enrolment, information on acute exacerbations was collected retrospectively through structured patient interviews and verified, whenever possible, against available hospital medical records. An acute exacerbation of COPD was defined as a worsening of respiratory symptoms beyond normal day-to-day variation that required treatment with systemic corticosteroids, antibiotics, or both, or resulted in hospitalisation, in accordance with the 2025 GOLD recommendations [2] and the ERS/ATS consensus statement [39].

For each participant, the total number of exacerbations experienced during the previous 12 months was recorded and used as the primary measure of exacerbation burden. Participants with two or more exacerbations during the preceding year were classified as frequent exacerbators, whereas those with fewer than two events were classified as infrequent exacerbators [8].

### 2.6. Laboratory Measurements

Venous blood samples were collected from all participants in the morning after an overnight fast of at least eight hours, before the administration of routine medication. Biochemical analyses were performed using serum obtained from blood collected in serum separator tubes and centrifuged at 3000 rpm for 10 min at room temperature. Serum samples were processed within two hours of collection and stored at −20 °C until analysis. Blood collected in EDTA tubes was used for haematological testing.

Haematological analyses included red blood cell count (RBC), haemoglobin concentration, white blood cell count (WBC), platelet count, absolute neutrophil and eosinophil counts, and eosinophil percentage, using standard automated laboratory methods. Fasting plasma glucose was measured by the glucose oxidase method. CRP concentrations were determined by immunoturbidimetric assay (mg/L), ESR by the Westergren method (mm/h), and fibrinogen by the Clauss coagulometric assay (mg/dL).

Serum TNF-α and IL-8 concentrations were measured using commercially available Quantikine enzyme-linked immunosorbent assay (ELISA) kits (R&D Systems, Minneapolis, MN, USA; Catalog Nos. DTA00D and D8000C, respectively), according to the manufacturer’s instructions. All samples were assayed in duplicate. The minimum detectable concentration was 6.23 pg/mL for TNF-α (assay range 15.6–1000 pg/mL) and 7.5 pg/mL for IL-8 (assay range 31.2–2000 pg/mL). Intra-assay and inter-assay coefficients of variation were below 10% for both assays. Results were expressed in pg/mL.

### 2.7. Statistical Analysis

For descriptive analyses, continuous variables were reported as mean ± standard deviation (SD) or median [interquartile range (IQR)], as appropriate. Group differences were evaluated using the independent samples *t*-test or the Mann–Whitney U test, according to data distribution assessed with the Shapiro–Wilk test. Categorical variables were presented as frequencies and percentages and compared using Pearson’s chi-square test or Fisher’s exact test. Spearman’s rank correlation was used to examine associations between inflammatory markers and spirometric parameters and between the inflammatory markers themselves. Because CRP, TNF-α, and IL-8 showed right-skewed distributions, natural logarithmic transformation was applied before multivariable analyses to improve model fit. ESR and fibrinogen were analysed per 10 mm/h and 100 mg/dL increment, respectively, to aid interpretation of effect estimates.

Patients were classified as having an eosinophilic phenotype if the peripheral blood eosinophil percentage was ≥2%, a threshold originally described in the context of acute exacerbations [40], and subsequently evaluated, alongside absolute eosinophil counts, in large stable-state COPD cohorts for association with future exacerbation risk [41]. As current evidence increasingly favours absolute counts over percentages, with 300 cells/μL identified as a clinically relevant threshold in this population [41], we additionally performed a sensitivity analysis using this absolute threshold.

For analyses requiring categorical status, elevated inflammatory marker levels were defined as CRP > 10 mg/L [12], ESR > 20 mm/h in men and >30 mm/h in women [42], and fibrinogen > 400 mg/dL [13]. CRP and fibrinogen cut-offs are consistent with values used in prior COPD cohort studies [12,13], whereas the ESR cut-off reflects general age- and sex-adjusted normal ranges [42], as no COPD-specific ESR threshold has been established in the literature [14]. These cut-offs were chosen for clinical interpretability rather than as COPD-specific thresholds. Dichotomisation was used only for the Kaplan–Meier survival analysis, whereas all regression-based analyses (negative binomial regression, GSEM, and the Cox proportional hazards model) modelled biomarkers as continuous variables.

Independent associations between inflammatory markers and annual exacerbation count were evaluated using negative binomial regression fitted to the full study population of COPD patients (n = 96). To assess the independent contribution of each inflammatory marker after progressive adjustment for potential confounders, three sequential regression models were fitted: Model 1 (univariable), Model 2 (adjusted for GOLD spirometric grade), and Model 3 (fully adjusted for GOLD grade, obesity, obstructive sleep apnoea syndrome, hypertension, and heart failure). Sensitivity analyses additionally incorporated age, sex, place of residence, smoking status and duration, diabetes mellitus, previous pulmonary tuberculosis, maintenance treatment regimen, and LTOT. Results are presented as incidence rate ratios (IRRs) with 95% confidence intervals (CIs) estimated with robust standard errors.

Generalised structural equation modelling (GSEM) was performed to examine the association between a latent systemic inflammation factor and annual exacerbation count. The latent factor was defined by five inflammatory biomarkers: log-transformed CRP (reference indicator; factor loading fixed at 1), ESR, fibrinogen, log-transformed TNF-α, and log-transformed IL-8. Annual exacerbation count was analysed using a Poisson model with a log link after adjustment for age, sex, and smoking duration. Effect estimates were presented as IRRs with 95% confidence intervals, using robust (Huber–White) standard errors.

Time to first exacerbation was analysed using Kaplan–Meier survival analysis. Differences between groups were assessed using the log-rank test after stratification according to elevated versus non-elevated levels of each inflammatory marker. A multivariable Cox proportional hazards model was fitted to determine which variables were independently associated with time to first exacerbation, with adjustment for tuberculosis history, smoking status, age (modelled as a quadratic term), sex, and BMI. The proportional hazards assumption was verified using Schoenfeld residuals. Results were expressed as hazard ratios (HR) with 95% confidence intervals. Tied event times were handled using the Breslow method.

All statistical analyses were performed using Stata 17.0 SE statistical software (StataCorp LLC, College Station, TX, USA). A *p*-value below 0.05 was considered statistically significant throughout.

## 3. Results

### 3.1. Baseline Characteristics of the Study Population

A total of 96 clinically stable patients with confirmed COPD across GOLD spirometric grades I to IV were enrolled in the study. Table 1 summarises the baseline characteristics of the study population, including demographic and clinical data, lung function parameters, inflammatory biomarkers, comorbidities, and health status assessment scores.

The mean age was 66.1 ± 9.2 years, and most participants were male (69.8%). Urban and rural residence were similarly represented (52.1% and 47.9%, respectively). The mean BMI was 27.2 ± 7.3 kg/m^2^. Current smokers accounted for 56.3% of the study population, with a mean smoking duration of 22.5 ± 21.8 years and a mean age at smoking initiation of 26.7 ± 8.7 years. A substantial proportion of patients (43.8%) were non-smokers: former smokers accounted for 16.7% of the cohort, and 27.1% had no documented smoking history.

Pulmonary function testing confirmed a predominantly severe obstructive pattern. The mean post-bronchodilator FEV_1_ was 42.7 ± 19.9% predicted, corresponding to GOLD III spirometric severity. Mean FVC was 77.5 ± 23.9% predicted, indicating a less pronounced reduction than that observed for FEV_1_. The mean FEV_1_/FVC ratio was 52.8 ± 13.2%, well below the diagnostic threshold of 70%, and the mean FEF_25–75_ was 29.2 ± 17.2% predicted. The relatively large standard deviations observed across spirometric parameters reflect the heterogeneity of the study cohort, which included patients from all GOLD spirometric grades (I–IV).

Cardiometabolic comorbidities were common in the study population. Arterial hypertension was the most common, affecting 68.8% of patients, followed by obesity (56.3%), diabetes mellitus (31.3%), and heart failure (28.1%). Other documented comorbidities included obstructive sleep apnoea syndrome (20.8%), atrial fibrillation (14.6%), and previous pulmonary tuberculosis (12.5%).

Symptom burden and health-related quality of life were substantially impaired across the cohort. The mean CAT score was 23.3 ± 8.8, indicating high symptom impact, and the mean mMRC grade was 2.5 ± 1.1, reflecting moderate-to-severe dyspnoea. The mean SGRQ-C total score was 51.4 ± 18.6, consistent with severely compromised health-related quality of life.

Regarding maintenance therapy, half of the patients received triple inhaled therapy (ICS/LABA/LAMA), 34.4% were treated with ICS/LABA, 8.3% received LABA monotherapy, and 7.3% received LABA/LAMA. Long-term oxygen therapy (LTOT) was prescribed in 31.3% of patients.

Systemic inflammatory markers were consistently elevated among the enrolled patients. Mean CRP concentration was 21.5 ± 17.7 mg/L, while mean fibrinogen and ESR values were 428.3 ± 120.4 mg/dL and 58.1 ± 21.0 mm/h. Mean serum concentrations of TNF-α and IL-8 were 13.7 ± 4.5 pg/mL and 33.8 ± 9.9 pg/mL, respectively.

### 3.2. Inflammatory Biomarkers, Pulmonary Function and Disease Severity

Inflammatory biomarker concentrations increased progressively across GOLD spirometric grades, as shown in Table 2. Mean CRP concentrations rose from 10.01 ± 5.12 mg/L in patients with GOLD grade I disease to 30.46 ± 21.19 mg/L in those with GOLD grade IV (*p* < 0.001). Similar patterns were observed for ESR, which rose from 37.52 ± 23.83 mm/h to 73.76 ± 18.26 mm/h (*p* < 0.001), TNF-α, which increased from 7.25 ± 3.45 pg/mL to 15.53 ± 4.13 pg/mL (*p* < 0.001), and IL-8, which increased from 17.19 ± 11.92 pg/mL to 37.33 ± 5.88 pg/mL (*p* < 0.001). Fibrinogen levels also increased across the severity spectrum, from 370.50 ± 85.05 mg/dL in GOLD I to 470.38 ± 108.49 mg/dL in GOLD IV (*p* = 0.026).

Spearman correlation analysis revealed significant inverse correlations between inflammatory markers and spirometric indices (Table 3). Among the biomarkers evaluated, ESR showed the strongest associations with pulmonary function, including FEV_1_ (r = −0.601), FVC (r = −0.548), FEV_1_/FVC ratio (r = −0.529), and FEF_25–75_ (r = −0.517). CRP correlations ranged from −0.316 (FEV_1_/FVC ratio) to −0.359 (FEV_1_ and FEF_25–75_). TNF-α associations were somewhat stronger, particularly for FEF_25–75_ (r = −0.416) and FEV_1_ (r = −0.394), with weaker values for FVC and the FEV_1_/FVC ratio. IL-8 correlated inversely with all four parameters (r = −0.292 to −0.366). Fibrinogen had the most attenuated associations overall (r = −0.220 to −0.232). All correlations reached statistical significance (*p* < 0.05).

Positive correlations were also identified among inflammatory biomarkers. The strongest relationships involved ESR, CRP, and fibrinogen, with correlation coefficients ranging from 0.682 to 0.778. IL-8 and TNF-α were moderately correlated (r = 0.500), whereas the remaining biomarker pairs showed weaker, but still significant, correlations (r = 0.322–0.454). Spirometric indices were strongly correlated with one another, with the highest coefficients observed between FEV_1_ and FVC (r = 0.922), followed by FEV_1_ and FEF_25–75_ (r = 0.879) and FEV_1_ and the FEV_1_/FVC ratio (r = 0.795).

### 3.3. Clinical Impact of Systemic Inflammation

Patient-reported outcome measures varied according to inflammatory marker status (Table 4). Patients with elevated CRP had higher CAT scores (23.4 vs. 14.9), mMRC grades (2.6 vs. 1.7), and SGRQ-C total scores (52.1 vs. 32.9) than those with normal CRP levels. Comparable findings were observed for IL-8, with higher CAT (25.1 vs. 15.2), mMRC (2.7 vs. 1.8), and SGRQ-C (54.2 vs. 34.1) scores among patients with elevated concentrations.

The most pronounced differences were observed for ESR and TNF-α. Patients with elevated ESR had markedly higher CAT (22.6 vs. 3.1), mMRC (2.5 vs. 0.5), and SGRQ-C scores (50.0 vs. 6.6) than those with normal ESR; however, these comparisons should be interpreted with caution given the marked subgroup imbalance for ESR (n = 2 vs. n = 94; Table 4). Likewise, elevated TNF-α levels were associated with higher CAT (25.6 vs. 16.8), mMRC (3.0 vs. 1.8), and SGRQ-C scores (59.6 vs. 35.9). All between-group comparisons were statistically significant (*p* < 0.001).

### 3.4. Eosinophilic COPD Phenotype

Patients were stratified into eosinophilic (EOS% ≥ 2%) and non-eosinophilic (EOS% < 2%) subgroups based on peripheral blood eosinophil percentage, yielding 40 and 56 patients, respectively. This threshold was selected on the basis of its established application in COPD clinical and translational research [40,43].

Median concentrations of all five inflammatory biomarkers were lower in the eosinophilic subgroup compared with non-eosinophilic patients. However, a statistically significant difference was observed only for ESR (47.5 [IQR 38.0–63.5] vs. 59.5 [50.3–70.9] mm/h; *p* = 0.040). Median CRP, fibrinogen, IL-8, and TNF-α values were also lower in eosinophilic patients, yet these differences were not statistically significant (all *p* > 0.05).

Lung function was better preserved in the eosinophilic subgroup, with significantly higher median FEV_1_ (49.5% [31.9–62.8] vs. 38.0% [24.0–49.5] predicted, *p* = 0.015) and FVC (86.5% [63.5–110.0] vs. 65.0% [55.5–87.2] predicted, *p* = 0.011).

Eosinophilic patients also reported a lower symptom burden and better health-related quality of life, reflected by lower CAT scores (19.0 [13.0–27.0] vs. 24.0 [19.0–32.0], *p* = 0.025), lower mMRC grades (2.0 [1.0–3.0] vs. 3.0 [2.0–4.0], *p* = 0.034), and lower SGRQ-C total scores (43.0 [32.0–60.0] vs. 52.5 [41.0–71.2], *p* = 0.025).

Annual exacerbation frequency and the prevalence of major comorbidities did not differ significantly between the two groups (all *p* > 0.05). Complete comparisons are presented in Table 5.

As a sensitivity analysis, patients were also classified using an absolute eosinophil count threshold of ≥300 cells/μL (n = 16 vs. n = 80). No statistically significant differences were observed for CAT (*p* = 0.247), mMRC (*p* = 0.745), SGRQ-C (*p* = 0.879), or annual exacerbation frequency (*p* = 0.182), likely reflecting reduced statistical power at this more restrictive threshold.

### 3.5. Systemic Inflammation and Exacerbation Burden

Among the 96 patients, 493 acute exacerbation events were reported during the preceding 12 months (mean ± SD, 5.14 ± 2.62; median [IQR], 5 [3,4,5,6,7]; range, 1–12), using the retrospective ascertainment approach previously described in the manuscript. Negative binomial regression was performed in the full cohort of 96 COPD patients to examine the associations with annual exacerbation frequency using sequentially adjusted models. The aim was to distinguish the total association of each inflammatory marker from its independent contribution after accounting for confounders. Some of these covariates, however, may lie on the causal pathway linking inflammation to exacerbation risk, rather than serving purely as adjustment variables.

In univariable analysis (Model 1), all five inflammatory markers were significantly associated with exacerbation frequency: CRP (IRR = 1.39, 95% CI: 1.25–1.54, *p* < 0.001), ESR (IRR = 2.29, 95% CI: 1.89–2.79, *p* < 0.001), fibrinogen (IRR = 1.61, 95% CI: 1.21–2.15, *p* < 0.001), TNF-α (IRR = 1.70, 95% CI: 1.35–2.14, *p* < 0.001), and IL-8 (IRR = 1.89, 95% CI: 1.54–2.33, *p* < 0.001).

After adjustment for GOLD grade (Model 2), CRP (IRR = 1.19, *p* = 0.002) and ESR (IRR = 1.67, *p* < 0.001) remained significantly associated with exacerbation frequency. The associations for fibrinogen and IL-8 were attenuated and approached statistical significance (both *p* = 0.091), whereas TNF-α was no longer statistically significant (*p* = 0.319).

Following full adjustment (Model 3), none of the inflammatory biomarkers showed an independent association with exacerbation frequency. Instead, GOLD grade (IRR = 1.34, *p* < 0.001), obesity (IRR = 1.20, *p* = 0.015), and heart failure (IRR = 1.34, *p* < 0.001) remained independently associated, whereas hypertension (IRR = 0.76, *p* < 0.001) and obstructive sleep apnoea syndrome (IRR = 0.59, *p* < 0.001) were associated with lower exacerbation rates. The fully adjusted model demonstrated good overall fit (Wald χ^2^ = 45.47, *p* < 0.001; pseudo-R^2^ = 0.597). The results are presented in Table 6.

Sensitivity analyses incorporating age, sex, place of residence, smoking status and duration, diabetes mellitus, atrial fibrillation, tuberculosis history, LTOT, and inhaled therapy regimen did not identify any additional factors independently associated with exacerbation frequency.

### 3.6. Time to First Exacerbation

Survival analysis included 96 patients with complete time-to-first-exacerbation data, contributing a total of 117.5 person-years of follow-up. The overall incidence rate of first exacerbation was 817.0 per 1000 person-years (95% CI: 668.9–998.0), reflecting the high exacerbation burden in the study population.

Kaplan–Meier survival analyses were performed after stratification according to serum TNF-α, IL-8, CRP, and fibrinogen status (Figure 1). For TNF-α, the survival curves for patients with normal and elevated concentrations showed minimal separation throughout the follow-up period. Elevated CRP and IL-8 levels were associated with shorter exacerbation-free survival compared to normal biomarker levels; however, these differences were not statistically significant after adjustment for sex, age group, and smoking status. Fibrinogen demonstrated the least pronounced difference between groups, with almost complete overlap of the survival curves throughout the follow-up period. Overall, none of the four inflammatory biomarkers were associated with a significant difference in exacerbation-free survival. Differences between groups were further evaluated using adjusted Cox proportional hazards models.

In the multivariable Cox proportional hazards model (Breslow method, robust standard errors) adjusted for tuberculosis history, smoking status, age (modelled as a quadratic term), sex, and BMI, biomarkers were modelled as continuous variables, consistent with their treatment in the main regression-based analyses.

Fibrinogen was the only biomarker reaching statistical significance (HR = 0.999 per mg/dL, 95% CI 0.997–1.000; *p* = 0.034); however, the per-unit effect size was negligible and the inverse direction of the association was contrary to the hypothesised pro-inflammatory trajectory, likely reflecting a scale artefact. CRP showed a borderline non-significant pattern (HR = 1.007 per mg/L, 95% CI 0.999–1.016; *p* = 0.090), while TNF-α (HR = 1.010 per pg/mL, 95% CI 0.973–1.049; *p* = 0.593) and IL-8 (HR = 1.006 per pg/mL, 95% CI 0.991–1.020; *p* = 0.461) were not significantly associated with time to first exacerbation.

Among the covariates, only the quadratic age term was statistically significant (HR = 0.9998, 95% CI 0.9997–0.9999; *p* = 0.007), consistent with a non-linear relationship between age and exacerbation timing. Tuberculosis history, smoking status, sex, and BMI were not independently associated with time to first exacerbation (all *p* > 0.05). The overall model was of borderline significance (Wald χ^2^_9_ = 15.26, *p* = 0.084). The results are summarised in Table 7.

### 3.7. Structural Equation Modelling of the Systemic Inflammatory Phenotype

A generalised structural equation model (GSEM) was fitted to examine the relationship between a latent systemic inflammation construct and COPD exacerbation frequency. The latent factor was identified by constraining the loading of log-transformed CRP to unity. All remaining inflammatory biomarkers loaded significantly onto the latent construct, including ESR (β = 33.42, SE = 3.22, z = 10.39, *p* < 0.001), fibrinogen (β = 152.50, SE = 16.37, z = 9.31, *p* < 0.001), log-transformed IL-8 (β = 0.433, SE = 0.094, z = 4.58, *p* < 0.001), and log-transformed TNF-α (β = 0.395, SE = 0.104, z = 3.78, *p* < 0.001). The estimated variance of the latent inflammation factor was 0.305 (SE = 0.062). Standardised factor loadings indicated that ESR had the strongest association with the latent construct (λ* = 0.881), followed by CRP (λ* = 0.771), fibrinogen (λ* = 0.703), IL-8 (λ* = 0.623), and TNF-α (λ* = 0.570).

The significant loadings of all five indicators support the construct validity of a unified systemic inflammatory phenotype encompassing the acute-phase response (CRP, fibrinogen, ESR) and pro-inflammatory cytokine signalling (IL-8, TNF-α). The measurement model estimates are presented in Table 8.

In the structural component of the GSEM, a one-unit increase in the latent inflammation factor was associated with a 40.7% higher exacerbation rate (IRR = 1.407, 95% CI 1.145–1.668, *p* < 0.001). Older age (IRR = 1.012 per year, 95% CI 1.001–1.023, *p* = 0.030), male sex (IRR = 1.232, 95% CI 1.036–1.428, *p* = 0.010), and longer smoking duration (IRR = 1.006 per year, 95% CI 1.002–1.010, *p* = 0.002) were also independently associated with increased exacerbation frequency. The structural model estimates are summarised in Table 9, and the corresponding path diagram and forest plot are shown in Figure 2.

Global fit indices (CFI, RMSEA, SRMR) are not estimable for GSEM with mixed response families in Stata 17. Local fit was assessed through indicator-level variance explained (R^2^): ESR = 0.777, CRP = 0.595, fibrinogen = 0.494, IL-8 = 0.388, TNF-α = 0.325. The model estimated 20 free parameters from 96 complete cases (ratio 4.8:1), which is below conventional thresholds; results should therefore be interpreted with appropriate caution and regarded as preliminary pending replication in a larger COPD cohort. Model convergence was robust: the log pseudolikelihood stabilised at −1324.857 by iteration 5, with no change at iteration 6.

As the 493 recorded exacerbations are clustered within 96 independent patients rather than representing independent observations, sample-size adequacy for the structural component was assessed using the ratio of independent observations to estimated parameters (96/20 = 4.8:1), as reported above, rather than the ratio of total events to predictors.

As a sensitivity analysis, GOLD spirometric grade was added to the structural component of the GSEM alongside age, sex, and smoking duration. The latent inflammatory construct remained significantly associated with exacerbation rate after this additional adjustment, although the effect was attenuated compared with the primary model (IRR = 1.186, 95% CI 1.020–1.379; *p* = 0.027 vs. IRR = 1.407 in the primary model). GOLD spirometric grade was also independently associated with higher exacerbation rates across all severity grades (grade II: IRR = 1.987, *p* = 0.010; grade III: IRR = 2.074, *p* = 0.005; grade IV: IRR = 2.674, *p* < 0.001). Further adjustment for the comorbidities included in the fully adjusted negative binomial model (obesity, OSAS, hypertension, and heart failure) was not performed because of the limited sample size and increased model complexity, which could compromise the stability and reliability of parameter estimates.

## 4. Discussion

The present study explored the relationship between systemic inflammation and multiple clinical outcomes of COPD using a range of complementary statistical approaches. Higher concentrations of inflammatory biomarkers corresponded to poorer pulmonary function, greater symptom burden, impaired health-related quality of life, and more advanced disease severity. While several biomarkers showed associations with exacerbation frequency in the initial analyses, these relationships weakened after adjustment for disease severity and major comorbidities. In contrast, the latent systemic inflammation factor derived from the GSEM remained independently associated with exacerbation frequency, indicating that the shared inflammatory signal across multiple biomarkers may capture information on exacerbation burden that is not retained by individual biomarkers after multivariable adjustment. By comparison, none of the individual inflammatory biomarkers showed a clinically meaningful association with time to first exacerbation in the multivariable survival analysis, with the exception of a negligible, likely artefactual inverse association for fibrinogen.

The study population, with a mean age of 66 years and predominantly male (69.8%), reflects the profile of patients with moderate-to-severe COPD typically encountered in tertiary respiratory care [44], closely matching the ECLIPSE [6] and COPDGene [45] cohorts, where disease severity and cumulative smoking exposure emerged as major drivers of systemic inflammation. Patients were drawn from both urban (52.1%) and rural (47.9%) settings, introducing additional variability in aetiological exposures beyond tobacco smoking. Rural residents in Eastern Europe may be exposed to biomass smoke and occupational irritants recognised as contributors to non-smoking COPD pathways [27], consistent with the substantial proportion of patients included in our study without a documented smoking history [46].

The mean BMI of 27.2 kg/m^2^ placed our study population in the overweight category, and more than half of the patients were classified as obese. The high prevalence of overweight and obesity in our cohort is consistent with the growing recognition that metabolic comorbidities frequently coexist with COPD [47]. Adipose tissue is metabolically active and secretes pro-inflammatory mediators, including TNF-α and IL-8, which may amplify the systemic inflammatory burden already driven by COPD itself [48], potentially explaining why obesity remained an independent correlate of exacerbation frequency in the fully adjusted negative binomial model.

The mean FEV_1_ of 42.7% predicted indicates a sample predominantly composed of individuals with GOLD spirometric grade III, a pattern commonly observed in hospital-based studies, where individuals with milder disease are less likely to be referred for specialist assessment [44]. Although this limits the generalisability of our findings to patients with milder COPD, it allowed us to evaluate the relationship between systemic inflammation and pulmonary function across a broad spectrum of clinically relevant disease severity.

The association between airflow limitation and systemic inflammation is well established in the COPD literature [6,49], and our results extend this evidence by demonstrating a progressive increase in all five inflammatory biomarkers across GOLD grades I to IV, although the strength of these associations differed. ESR showed the strongest correlations with spirometric indices, whereas fibrinogen showed the weakest, possibly reflecting ESR’s role as a non-specific acute-phase marker of cumulative inflammatory burden rather than any single inflammatory pathway. Inverse correlations with spirometric indices were also observed, most notably with FEF_25–75_, a sensitive marker of small-airway dysfunction previously linked to persistent inflammation even in the absence of marked FVC changes [50,51].

Patient-reported outcome measures indicated a substantial clinical burden, with CAT, mMRC, and SGRQ-C scores consistent with severe symptoms [52], clinically relevant dyspnoea [34], and impaired health-related quality of life [36], in line with the predominance of GOLD spirometric grade III–IV disease in our sample.

The high symptom burden observed here is not solely explained by the degree of airflow limitation: patients with elevated inflammatory biomarker concentrations reported worse symptom burden and poorer health-related quality of life, reinforcing the concept that systemic inflammation contributes to the clinical expression of COPD independently of spirometric severity [53]. These dichotomised comparisons should be interpreted with appropriate caution, however, as subgroup sizes were markedly imbalanced for some biomarkers, most notably ESR (n = 2 vs. n = 94), reflecting the near-universal elevation of ESR in this advanced COPD population. The primary analyses (negative binomial regression, GSEM, Cox model) modelled biomarkers as continuous variables and are not subject to this limitation.

The high prevalence of cardiovascular and metabolic comorbidities in our cohort reflects the complex multisystem nature of COPD, with conditions such as hypertension, obesity, diabetes mellitus, and heart failure frequently coexisting with chronic airflow obstruction [54]. These conditions may influence circulating inflammatory biomarker levels through shared pathways, including systemic inflammation, oxidative stress, and endothelial dysfunction [29,55], supporting their inclusion as covariates in the multivariable analyses. Adjustment for major comorbidities attenuated the associations between individual inflammatory biomarkers and exacerbation frequency. However, disease severity, obesity, and heart failure remained independently associated with exacerbations, indicating that the clinical course of COPD is shaped by both pulmonary disease severity and common extrapulmonary comorbidities.

Hypertension and diabetes mellitus were among the most frequent comorbidities. Although both conditions have been linked to chronic low-grade systemic inflammation and increased circulating inflammatory biomarkers [29,55], diabetes did not remain independently associated with exacerbation frequency after multivariable adjustment, whereas hypertension showed an unexpected inverse association, discussed further below.

Heart failure was one of the most clinically relevant comorbidities. Its persistence as an independent correlate of exacerbation frequency is consistent with the recognised role of heart failure as a major determinant of adverse outcomes in COPD and highlights the importance of accounting for this comorbidity in biomarker studies [56].

One unexpected finding in our regression model was the inverse association between OSAS and exacerbation frequency (IRR = 0.59, *p* < 0.001). Although COPD-OSA overlap syndrome is generally associated with worse outcomes when untreated, adherence to continuous positive airway pressure (CPAP) therapy has been shown to reduce exacerbations, hospitalisations, and mortality to levels comparable with COPD alone [57]. As CPAP adherence was not captured in our dataset, we cannot determine whether this protective association reflects effective CPAP treatment in our OSAS patients or another underlying mechanism; this warrants further investigation in future prospective studies. As with the hypertension finding, this association should also be interpreted cautiously given the sample size relative to model complexity.

Previous pulmonary tuberculosis, identified in 12.5% of patients, reflects the continuing clinical relevance of post-tuberculosis lung disease in high-burden settings such as Romania [30]. Post-tuberculosis lung disease is increasingly recognised as a distinct chronic respiratory condition characterised by persistent structural lung abnormalities and ongoing inflammatory changes that may contribute to the clinical heterogeneity of COPD and influence systemic inflammatory biomarker profiles [58]. Although not independently associated with exacerbation frequency in our analyses, its prevalence warrants consideration as a potential phenotype modifier in settings where tuberculosis remains endemic.

Another interesting observation was the correlation pattern among the inflammatory biomarkers themselves. ESR, CRP, and fibrinogen were strongly intercorrelated, consistent with their shared role as acute-phase reactants, whereas TNF-α and IL-8 showed more moderate associations. This indicates that the biomarkers capture complementary rather than identical components of the systemic inflammatory response. The observed interrelationships support a multidimensional approach to modelling systemic inflammation rather than relying on individual biomarkers. The GSEM enabled the common inflammatory signal shared across multiple biomarkers to be evaluated as a latent construct, providing a more integrated representation of systemic inflammation in COPD.

Patients with an eosinophilic phenotype (EOS% ≥ 2%) demonstrated better preserved pulmonary function and a lower symptom burden, with significantly higher FEV_1_ and FVC and significantly lower CAT, mMRC, and SGRQ-C scores than those without peripheral eosinophilia, although the relationship between blood and sputum eosinophils in COPD remains the subject of debate. Among the inflammatory biomarkers, only ESR differed significantly between phenotypes. This may reflect the relatively small subgroup sizes rather than the absence of biological differences, a possibility reinforced by the ≥300 cells/μL sensitivity analysis, which yielded an even smaller subgroup and more imbalanced eosinophilic subgroup, and no significant differences observed for any outcome. Exacerbation frequency and comorbidity burden were comparable between phenotypes, indicating that the observed differences were primarily related to pulmonary function and symptom burden rather than overall disease complexity. Treatment response to inhaled corticosteroids, for which blood eosinophils are a recognised guide [43], was beyond the scope of the present study.

The sequential-adjustment negative binomial regression revealed a pattern that is biologically coherent with the presented structural equation model. Associations between individual biomarkers and exacerbation frequency progressively weakened after adjustment for GOLD spirometric grade and comorbidities, suggesting that much of their apparent effect is captured by disease severity and comorbid burden rather than reflecting an independent contribution. One plausible explanation is that inflammation influences exacerbation risk not in isolation, but through its associations with airflow limitation severity, obesity, sleep-disordered breathing, hypertension, and cardiac dysfunction. In this context, these comorbidities may lie, at least in part, on the causal pathway between inflammation and exacerbation risk, although this could not be formally tested with the present cross-sectional design.

Obesity remained independently linked to exacerbation frequency in the fully adjusted model (IRR = 1.20, *p* = 0.015), fitting with evidence that adipose tissue releases pro-inflammatory mediators, including TNF-α and IL-8, that could add to the inflammatory burden already present in COPD [48]. GOLD spirometric grade, however, showed the strongest independent relationship with exacerbation frequency overall (IRR = 1.34, *p* < 0.001). This is a reminder of just how central airflow limitation severity is to exacerbation risk [7,8].

In the multivariable Cox proportional hazards model, most inflammatory biomarkers were not independently associated with time to first exacerbation. Fibrinogen was the only biomarker reaching statistical significance when modelled as a continuous variable (HR = 0.999 per mg/dL, *p* = 0.034). However, the very small per-unit effect and the inverse direction of the association, contrary to the expected relationship between higher fibrinogen levels and earlier exacerbation, suggest that this finding may be related to the biomarker scale and should therefore be interpreted cautiously. CRP showed a borderline, non-significant association (*p* = 0.090). Among the covariates, the quadratic age term was the only variable independently associated with time to first exacerbation, indicating a non-linear relationship between age and exacerbation timing that was not explained by inflammatory biomarker levels.

This mismatch between the count-based and time-to-event results is not necessarily a contradiction. Systemic inflammation may be more closely related to the overall burden of recurrent exacerbations than to the timing of the initial event. This interpretation is consistent with the stronger associations observed in the count-based analyses and the GSEM, both of which capture cumulative exacerbation burden rather than the occurrence of a single event. The high incidence of time-to-first-exacerbation events (817.0 per 1000 person-years) in our study group may also have reduced the ability of the survival analysis to discriminate between inflammatory phenotypes.

An important strength of our study was the use of GSEM to model systemic inflammation as a latent construct rather than evaluating each biomarker separately. All five inflammatory markers loaded significantly onto the latent factor (all *p* < 0.001), indicating that they captured a shared dimension of the inflammatory response despite reflecting different biological processes. This approach acknowledges the multifaceted nature of systemic inflammation in COPD and avoids reliance on individual biomarkers.

The GSEM analysis showed that the latent inflammatory factor was independently associated with a 40.7% increase in the expected annual exacerbation rate per unit increase (IRR = 1.407, 95% CI 1.145–1.668, *p* < 0.001), even after adjustment for age, smoking duration, sex, and other variables, indicating that the latent inflammatory construct is associated with exacerbation risk beyond established clinical factors. These observations suggest that multidimensional inflammatory profiling, rather than any individual circulating biomarker, may better capture clinically relevant inflammatory burden.

This finding aligns with the underlying biological mechanisms, because systemic inflammation in COPD arises from multiple interacting inflammatory pathways. Acute-phase reactants and circulating cytokines reflect complementary aspects of this response, and modelling their shared variance may therefore provide a more robust assessment of inflammatory burden than analysing each marker individually. The pattern of standardised loadings further clarifies the relative contribution of each biomarker: the acute-phase reactants (ESR, CRP, fibrinogen) showed stronger alignment with the latent construct (λ = 0.703–0.881) than the cytokines (λ = 0.570–0.623), consistent with greater within-patient stability of acute-phase proteins compared with the higher biological variability of circulating cytokines measured at a single time point. Although this approach requires external validation, it may offer a useful framework for future studies investigating inflammatory phenotypes and risk stratification in COPD.

The attenuation observed when the GSEM was additionally adjusted for GOLD spirometric grade suggests that part of the association of the latent construct with exacerbation rate overlaps with disease severity. Given the relatively low observations-to-parameters ratio in both the primary and sensitivity models, this ratio should be interpreted cautiously, with model convergence, standard errors, and indicator-level R^2^ providing additional evidence of estimation stability. Full adjustment for the comorbidity set included in the fully adjusted negative binomial model was not performed given the limited sample size and increased model complexity. Accordingly, the latent construct and the individual biomarkers were evaluated under different adjustment sets, and direct comparisons between these analytical approaches should be interpreted as exploratory.

From a clinical perspective, our results highlight the importance of considering systemic inflammation alongside established clinical characteristics when assessing patients with COPD. Although the individual inflammatory biomarkers did not retain independent associations with exacerbation frequency after multivariable adjustment, the latent inflammatory construct remained independently associated with exacerbation burden, supporting the potential value of a multidimensional approach to systemic inflammation. Further prospective, multicentre studies are needed to validate these findings and determine whether this approach adds clinically meaningful information to conventional COPD assessment.

### Limitations

Several limitations should be acknowledged. The cross-sectional design precludes conclusions regarding the temporal relationship between systemic inflammation and exacerbations. Consequently, it remains unclear whether systemic inflammation precedes exacerbations or primarily reflects more advanced disease and cumulative exacerbation burden. In addition, exacerbation history was assessed retrospectively through patient interview, which may be subject to recall bias, particularly for milder exacerbations not requiring hospitalisation.

The predominance of patients with advanced COPD reflects the tertiary-care setting in which the study was conducted and may limit the applicability of the findings to patients with milder COPD managed in primary care. Some subgroup analyses, particularly the comparison between eosinophilic and non-eosinophilic patients, may have been underpowered to detect modest differences in inflammatory biomarker concentrations; therefore, non-significant findings should be interpreted cautiously.

Inflammatory biomarkers were measured only once during a period of clinical stability and therefore could not capture temporal fluctuations in systemic inflammation. Also, a history of pulmonary tuberculosis was present in 12.5% of the cohort, which may have contributed to clinical and inflammatory heterogeneity.

Finally, although robust standard errors were used, the structural component of the GSEM relied on a Poisson specification and may not have fully accounted for all sources of residual confounding. Furthermore, the number of estimated parameters relative to the sample size was substantial, increasing the potential for model overfitting. Accordingly, the GSEM findings should be interpreted cautiously and require confirmation in larger, independent COPD studies.

## 5. Conclusions

This study characterised the systemic inflammatory profile of a hospital-based COPD cohort using five circulating biomarkers (CRP, ESR, fibrinogen, TNF-α, and IL-8) across complementary analytical approaches. Systemic inflammatory activity increased with disease severity, was associated with impaired lung function, greater symptom burden, and poorer health-related quality of life, and was linked to a higher exacerbation burden in the unadjusted analyses.

Although the associations between individual biomarkers and exacerbation frequency were attenuated after adjustment for disease severity and major comorbidities, a latent inflammatory construct derived from all five biomarkers using generalised structural equation modelling remained independently associated with exacerbation rate, including after additional adjustment for GOLD spirometric grade. By contrast, neither the individual biomarkers nor the eosinophilic phenotype showed a clinically meaningful association with time to first exacerbation.

Overall, these findings support the value of integrating multiple inflammatory biomarkers within a latent-variable framework rather than considering individual markers in isolation. Future prospective multicentre studies should determine whether this multidimensional approach translates into improved inflammatory phenotyping and clinical risk stratification in patients with COPD.

## Figures and Tables

**Figure 1:**
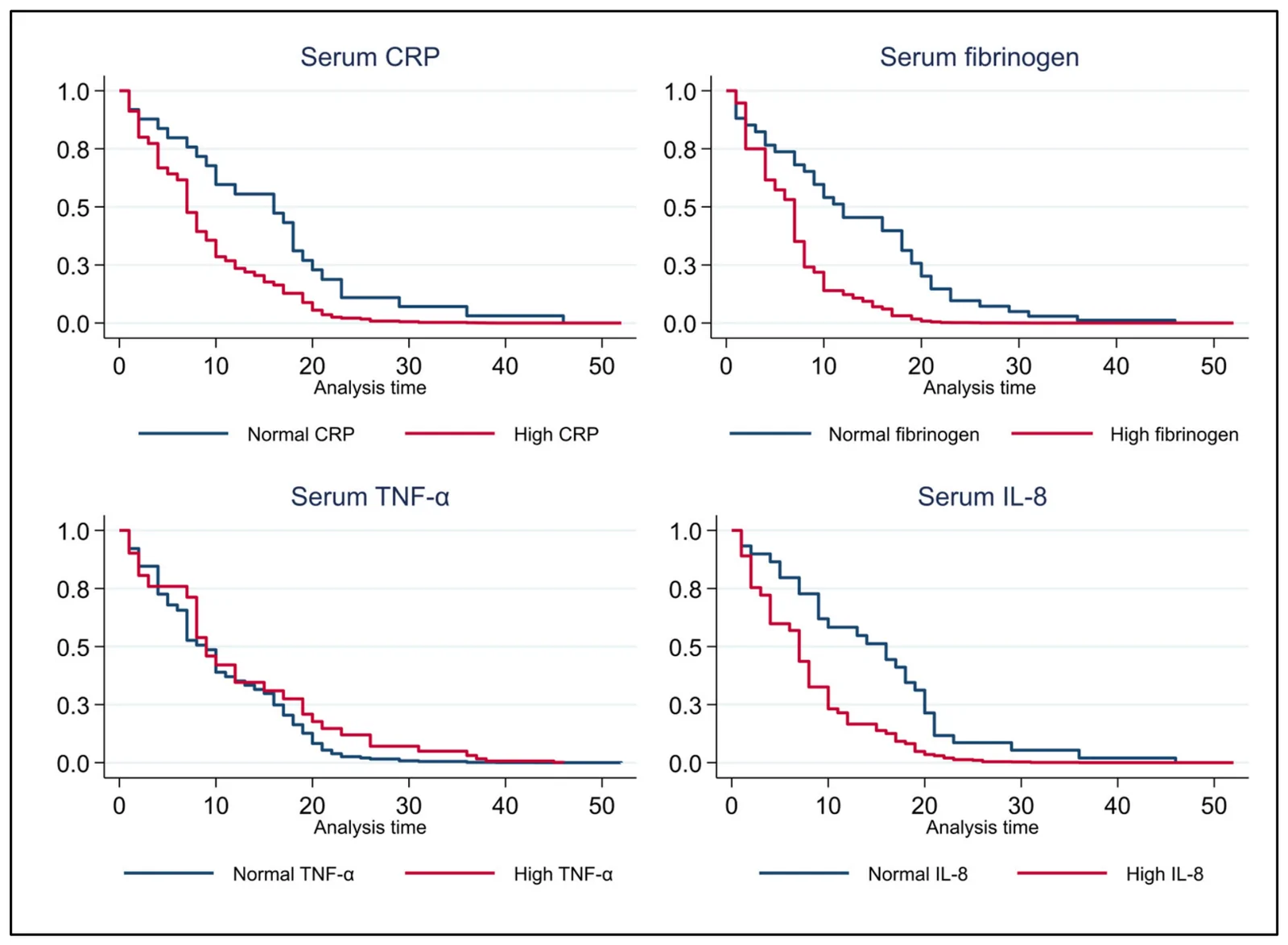
Kaplan–Meier curves for time to first COPD exacerbation according to serum CRP, fi-brinogen, TNF-α, and IL-8 status. Blue lines indicate patients with normal biomarker levels, whereas red lines indicate patients with elevated biomarker levels. Analysis time is expressed in months. CRP: C-reactive protein; TNF-α: tumour necrosis factor-α; IL-8, interleukin-8.

**Figure 2:**
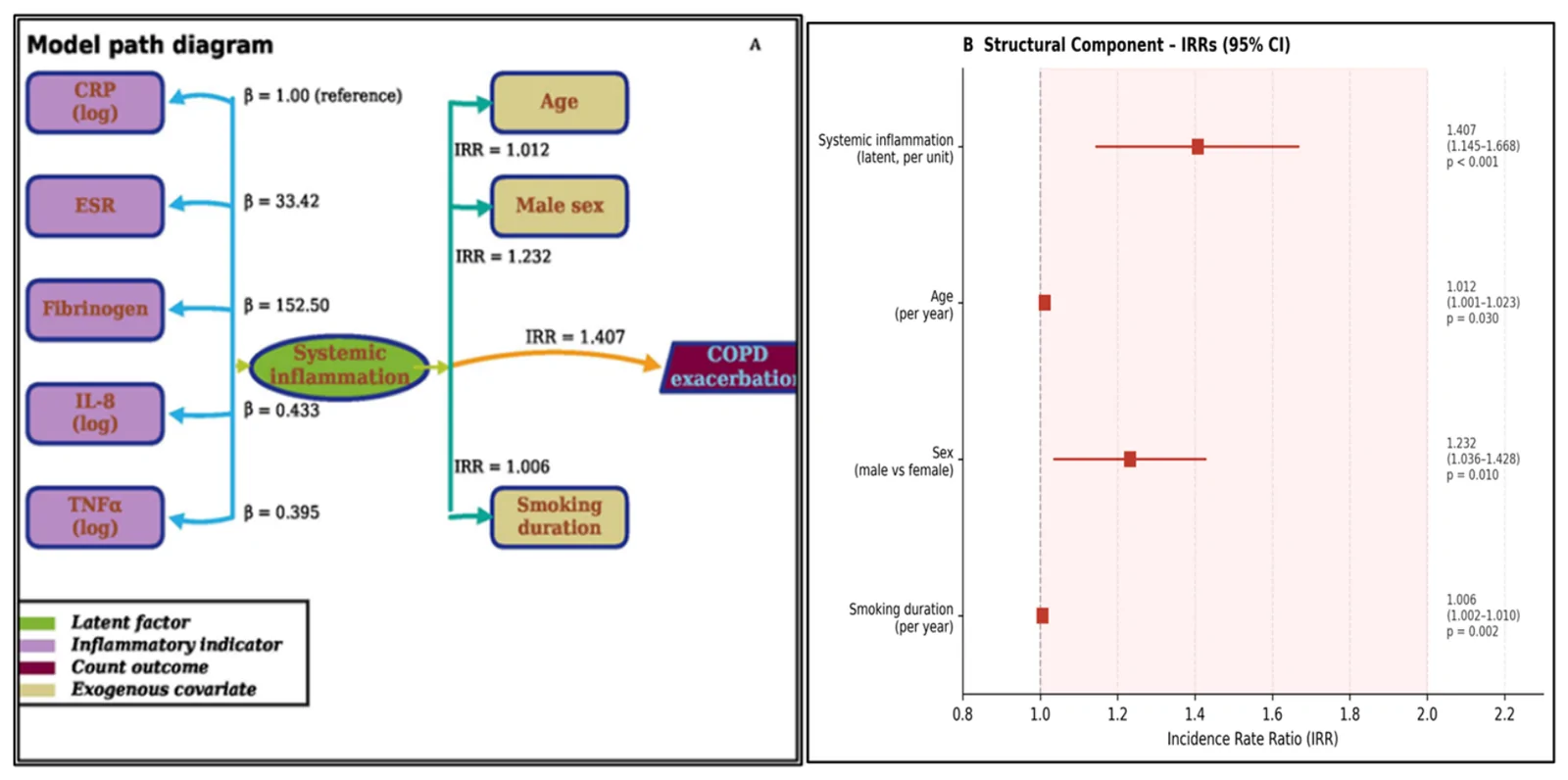
GSEM of the association between systemic inflammation and COPD exacerbation frequency. (**A**) Path diagram illustrating the latent systemic inflammation construct, its observed inflammatory indicators, and the structural associations with COPD exacerbation frequency. (**B**) Forest plot of incidence rate ratios (IRRs) and 95% confidence intervals (95% CIs) for variables included in the structural model. CRP: C-reactive protein; ESR: erythrocyte sedimentation rate; IL-8: interleukin-8; TNF-α: tumour necrosis factor-α.

**Table 1:** Baseline characteristics of the study population (n = 96).

DEMOGRAPHICS			SYMPTOM BURDEN AND QUALITY OF LIFE		
	Mean	SD		Mean	SD
AGE (YEARS)	66.06	9.18	CAT score	23.32	8.76
BMI (KG/M2)	27.24	7.31	mMRC grade	2.52	1.10
MALE SEX	69.79%	—	SGRQ-C score	51.39	18.57
URBAN RESIDENCE	52.08%	—			
SMOKING HISTORY			Comorbidities		
CURRENT SMOKER	56.25%	—	Heart failure	28.13%	—
SMOKING DURATION (YEARS)	22.52	21.75	Hypertension	68.75%	—
AGE AT SMOKING ONSET (YEARS)	26.68	8.68	Diabetes mellitus	31.25%	—
PULMONARY FUNCTION			Atrial fibrillation	14.58%	—
FEV1 (% PREDICTED)	42.73	19.92	Obesity	56.25%	—
FVC (% PREDICTED)	77.47	23.89	Tuberculosis history	12.50%	—
FEF25–75 (% PREDICTED)	29.16	17.21	OSAS	20.83%	—
FEV1/FVC RATIO	52.84	13.24			
MAINTENANCE THERAPY			Inflammation markers		
LABA MONOTHERAPY	8.3%	—	CRP (mg/L)	21.46	17.70
LABA/LAMA	7.3%	—	Fibrinogen (mg/dL)	428.28	120.36
ICS/LABA	34.4%	—	ESR (mm/h)	58.08	21.04
ICS/LABA/LAMA (TRIPLE THERAPY)	50%	—	TNF-α (pg/mL)	13.67	4.51
LTOT	31.3%	—	IL-8 (pg/mL)	33.80	9.88

Continuous variables are presented as mean ± standard deviation (SD), and categorical variables as percentages. BMI: body mass index; FEV_1_: forced expiratory volume in the first second; FVC: forced vital capacity; FEF_25–75_: forced expiratory flow between 25% and 75% of FVC; CAT: COPD Assessment Test; mMRC: modified Medical Research Council dyspnoea scale; SGRQ-C: St George’s Respiratory Questionnaire for COPD; OSAS: obstructive sleep apnoea syndrome; LABA: long-acting beta-agonist; LAMA: long-acting muscarinic antagonist; ICS: inhaled corticosteroid; LTOT: long-term oxygen therapy; CRP: C-reactive protein; ESR: erythrocyte sedimentation rate; TNF-α: tumour necrosis factor α; IL-8: interleukin-8.

**Table 2:** Inflammatory biomarker concentrations according to GOLD spirometric grade (n = 96).

	GOLD I (n = 6)	GOLD II (n = 20)	GOLD III (n = 38)	GOLD IV (n = 32)	*p* Value
CRP (mg/L)	10.01 ± 5.12	15.57 ± 11.53	18.80 ± 15.46	30.46 ± 21.19	<0.001
Fibrinogen (mg/dL)	370.50 ± 85.05	398.35 ± 113.78	417.71 ± 130.52	470.38 ± 108.49	0.026
ESR (mm/h)	37.52 ± 23.83	46.04 ± 14.43	54.47 ± 17.36	73.76 ± 18.26	<0.001
TNF-α (pg/mL)	7.25 ± 3.45	10.65 ± 3.61	14.70 ± 3.73	15.53 ± 4.13	<0.001
IL-8 (pg/mL)	17.19 ± 11.92	27.16 ± 11.72	36.94 ± 6.54	37.33 ± 5.88	<0.001

Data are presented as mean ± standard deviation (SD). *p* values were calculated using the Kruskal–Wallis test. GOLD: Global Initiative for Chronic Obstructive Lung Disease; CRP: C-reactive protein; ESR: erythrocyte sedimentation rate; TNF-α: tumour necrosis factor-α; IL-8: interleukin-8.

**Table 3:** Spearman correlation matrix of inflammatory markers and spirometric parameters in COPD patients.

	IL-8	TNF-α	ESR	CRP	Fibrinogen	FEV_1_	FVC	FEV_1_/FVC	FEF_25–75_
IL-8	1								
TNF-α	0.5	1							
ESR	0.454	0.405	1						
CRP	0.322	0.335	0.778	1					
Fibrinogen	0.366	0.364	0.682	0.729	1				
FEV_1_	−0.366	−0.394	−0.601	−0.359	−0.226	1			
FVC	−0.329	−0.343	−0.548	−0.349	−0.232	0.922	1		
FEV_1_/FVC	−0.292	−0.337	−0.529	−0.316	−0.232	0.795	0.542	1	
FEF_25–75_	−0.331	−0.416	−0.517	−0.359	−0.220	0.879	0.788	0.747	1

Values are presented as Spearman correlation coefficients (r). All correlations were statistically significant (*p* < 0.05). CRP: C-reactive protein; ESR: erythrocyte sedimentation rate; TNF-α: tumour necrosis factor α; IL-8, interleukin-8; FEV_1_, forced expiratory volume in one second; FVC, forced vital capacity; FEF_25–75_, forced expiratory flow between 25% and 75% of forced vital capacity.

**Table 4:** Patient-reported outcome measures according to CRP, IL-8, ESR, and TNF-α status.

Clinical Score	Group	CRP (n = 26, 70)	IL-8 (n = 30, 66)	ESR (n = 2, 94)	TNF-α (n = 65, 31)
CAT	Normal	14.9 ± 11.7	15.2 ± 12.0	3.1 ± 3.6	16.8 ± 11.8
	Elevated	23.4 ± 9.2	25.1 ± 8.6	22.6 ± 9.7	25.6 ± 8.6
	*p* value	<0.001	<0.001	<0.001	<0.001
mMRC	Normal	1.7 ± 1.3	1.8 ± 1.2	0.5 ± 0.5	1.8 ± 1.2
	Elevated	2.6 ± 1.1	2.7 ± 1.1	2.5 ± 1.1	3.0 ± 0.9
	*p* value	<0.001	<0.001	<0.001	<0.001
SGRQ-C	Normal	32.9 ± 26.9	34.1 ± 25.8	6.6 ± 7.6	35.9 ± 25.1
	Elevated	52.1 ± 18.3	54.2 ± 17.8	50.0 ± 20.3	59.6 ± 15.1
	*p* value	<0.001	<0.001	<0.001	<0.001

Data are presented as mean ± standard deviation (SD). CAT: COPD Assessment Test; mMRC: modified Medical Research Council dyspnoea scale; SGRQ-C: St George’s Respiratory Questionnaire for COPD; CRP: C-reactive protein; IL-8: interleukin-8; ESR: erythrocyte sedimentation rate; TNF-α: tumour necrosis factor α; n = normal/elevated subgroup size for each biomarker.

**Table 5:** Clinical and inflammatory characteristics according to eosinophilic phenotype (EOS ≥ 2% vs. EOS < 2%) in COPD patients.

Variable	EOS-High (n = 40) Median [IQR]	EOS-Low (n = 56) Median [IQR]	*p* Value
CRP (mg/L)	12.5 [8.9–23.7]	18.3 [11.0–29.8]	0.144
Fibrinogen (mg/dL)	381.0 [313.2–492.0]	420.5 [390.5–522.0]	0.052
ESR (mm/h)	47.5 [38.0–63.5]	59.5 [50.3–70.9]	0.040
IL-8 (pg/mL)	34.3 [24.9–40.3]	35.4 [30.8–41.0]	0.448
TNF-α (pg/mL)	12.5 [9.2–16.2]	14.2 [11.5–16.2]	0.363
Lung function			
FEV_1_ (% predicted)	49.5 [31.9–62.8]	38.0 [24.0–49.5]	0.015
FVC (% predicted)	86.5 [63.5–110.0]	65.0 [55.5–87.2]	0.011
Symptom burden and quality of life			
CAT score	19.0 [13.0–27.0]	24.0 [19.0–32.0]	0.025
mMRC grade	2.0 [1.0–3.0]	3.0 [2.0–4.0]	0.034
SGRQ-C total score	43.0 [32.0–60.0]	52.5 [41.0–71.2]	0.025
Exacerbations			
Exacerbation frequency (n/year)	4.0 [3.2–6.0]	5.0 [3.0–8.0]	0.421
Comorbidities, %			
Diabetes mellitus	34.2%	30.4%	0.694
Hypertension	65.8%	69.6%	0.694
Heart failure	28.9%	25.0%	0.671
Atrial fibrillation	15.8%	14.3%	0.841
OSAS	21.1%	19.6%	0.867

Data are presented as median [interquartile range (IQR)] for continuous variables and percentages for categorical variables. EOS: eosinophils; CRP: C-reactive protein; ESR: erythrocyte sedimentation rate; IL-8: interleukin-8; TNF-α: tumour necrosis factor-α; FEV_1_: forced expiratory volume in one second; FVC: forced vital capacity; CAT: COPD Assessment Test; mMRC: modified Medical Research Council dyspnoea scale; SGRQ-C: St George’s Respiratory Questionnaire for COPD; OSAS: obstructive sleep apnoea syndrome.

**Table 6:** Sequentially adjusted negative binomial regression models for factors associated with annual COPD exacerbation frequency.

Variable	Model 1 (Univariable) IRR (95% CI), *p*	Model 2 (GOLD-Adjusted) IRR (95% CI), *p*	Model 3 (Fully Adjusted) IRR (95% CI), *p*
CRP (log, mg/L)	1.39 (1.25–1.54), <0.001	1.19 (1.07–1.33), 0.002	1.03 (0.91–1.16), 0.657
ESR (per 10 mm/h)	2.29 (1.89–2.79), <0.001	1.67 (1.29–2.17), <0.001	1.14 (0.79–1.63), 0.486
Fibrinogen (per 100 mg/dL)	1.61 (1.21–2.15), <0.001	1.25 (0.96–1.61), 0.091	0.99 (0.73–1.36), 0.965
TNF-α (log, pg/mL)	1.70 (1.35–2.14), <0.001	1.15 (0.87–1.51), 0.319	0.96 (0.76–1.22), 0.765
IL-8 (log, pg/mL)	1.89 (1.54–2.33), <0.001	1.27 (0.96–1.66), 0.091	1.06 (0.77–1.46), 0.739
Model 3 covariates			
GOLD grade	—	—	1.34 (1.18–1.54), <0.001
Obesity	—	—	1.20 (1.04–1.38), 0.015
OSAS	—	—	0.59 (0.47–0.75), <0.001
Hypertension	—	—	0.76 (0.67–0.87), <0.001
Heart failure	—	—	1.34 (1.15–1.58), <0.001

Model 1: univariable model (each inflammatory biomarker entered separately). Model 2: adjusted for GOLD grade. Model 3: additionally adjusted for obesity, obstructive sleep apnoea syndrome (OSAS), hypertension, and heart failure. Results are presented as incidence rate ratios (IRRs) with 95% confidence intervals (CIs). CRP: C-reactive protein; ESR: erythrocyte sedimentation rate; TNF-α: tumour necrosis factor-α; IL-8: interleukin-8; OSAS: obstructive sleep apnoea syndrome; GOLD: Global Initiative for Chronic Obstructive Lung Disease.

**Table 7:** Multivariable Cox proportional hazards model for time to first COPD exacerbation, with inflammatory biomarkers modelled as continuous variables.

Variable	HR	95% CI	*p* Value
CRP (per mg/L)	1.007287	0.999–1.016	0.090
Fibrinogen (per mg/dL)	0.9986398	0.997–1.000	0.034
TNF-α (per pg/mL)	1.010318	0.973–1.049	0.593
IL-8 (per pg/mL)	1.005524	0.991–1.020	0.461
Tuberculosis history	0.7818058	0.557–1.098	0.155
Current smoker	1.18908	0.867–1.631	0.282
Age (quadratic term)	0.9998	0.9997–0.9999	0.007
Male sex	0.8335791	0.589–1.179	0.303
BMI	1.005236	0.984–1.027	0.632

Results are presented as hazard ratios (HRs) with 95% confidence intervals (95% CIs). CRP: C-reactive protein; TNF-α: tumour necrosis factor-α; IL-8: interleukin-8; BMI: body mass index.

**Table 8:** Measurement model of the latent systemic inflammation construct (GSEM).

Indicator	Factor Loading (β)		*SE*	*z*	*p*-Value
	Raw β	λ*			
CRP (log)	1.000	0.771	—	—	Reference
ESR	33.424	0.881	3.216	10.39	<0.001
Fibrinogen	152.495	0.703	16.373	9.31	<0.001
IL-8 (log)	0.433	0.623	0.094	4.58	<0.001
TNF-α (log)	0.395	0.570	0.104	3.78	<0.001

SE: standard error; λ*: standardised factor loading; CRP: C-reactive protein; ESR: erythrocyte sedimentation rate; IL-8: interleukin-8; TNF-α: tumour necrosis factor-α.

**Table 9:** Structural model estimates from the GSEM of COPD exacerbation frequency.

Variable	β	SE	z	IRR	95% CI	*p* Value
Age (per year)	0.012	0.006	2.18	1.012	1.001–1.023	0.030
Male sex	0.209	0.081	2.57	1.232	1.036–1.428	0.010
Smoking duration (per year)	0.006	0.002	3.06	1.006	1.002–1.010	0.002
Latent systemic inflammation	0.341	0.095	3.60	1.407	1.145–1.668	<0.001

Annual exacerbation count was modelled using a Poisson distribution with a log link. Robust (Huber–White) standard errors were applied. IRR: incidence rate ratio; CI: confidence interval; SE: standard error.

## Data Availability

The data presented in this study are available upon request from the corresponding author. The data are not publicly available due to the patient personal data protection policy of the university and hospital.
